# Patient specific selection of lateral wall cochlear implant electrodes based on anatomical indication ranges

**DOI:** 10.1371/journal.pone.0206435

**Published:** 2018-10-26

**Authors:** Max Eike Timm, Omid Majdani, Tobias Weller, Mayra Windeler, Thomas Lenarz, Andreas Büchner, Rolf Benedikt Salcher

**Affiliations:** Cluster of Excellence Hearing4all, Department of Otorhinolaryngology, Hannover Medical School, Hannover, Lower Saxony, Germany; University of California Irvine, UNITED STATES

## Abstract

**Objectives:**

The aim of this study was to identify anatomical indication ranges for different lateral wall cochlear implant electrodes to support surgeons in the preoperative preparation.

**Methods:**

272 patients who were implanted with a FLEX^20,^ FLEX^24^, FLEX^28^, or a custom-made device (CMD) were included in this study. The cochlear duct length (*CDL*) and basal cochlear diameter (length *A*) were measured within preoperative imaging data. The parameter *A* was then employed to additionally compute *CDL* estimates using literature approaches. Moreover, the inserted electrode length (*IEL*) and insertion angle (*IA*) were measured in postoperative CT data. By combining the preoperative measurements with the *IA* data, the covered cochlea length (*CCL*) and relative cochlear coverage (*CC*) were determined for each cochlea.

**Results:**

The measurements of the *CDL* show comparable results to previous studies. While *CDL* measurements and estimations cover similar ranges overall, severe deviations occur in individual cases. The electrode specific *IEL* and *CCL* are fairly consistent and increase with longer electrodes, but relatively wide ranges of electrode specific *CC* values were found due to the additional dependence on the respective *CDL*. Using the correlation of *IEL* and *CCL* across electrode arrays, *CDL* ranges for selected arrays were developed (FLEX^24^: 31.3–34.4, FLEX^28^: 36.2–40.1, FLEX^Soft^: 40.6–44.9).

**Conclusions:**

Our analysis shows that electrode specific *CC* varies due to the *CDL* variation. Preoperative measurement of the *CDL* allows for an individualized implant length selection yielding optimized stimulation and a reduced risk of intraoperative trauma. The *CDL*, as derived from preoperative CT imaging studies, can help the implant surgeon select the appropriate electrode array to maximize the patient’s outcomes.

## Introduction

Cochlear implantation is a technology for patients with total, severe or frequency specific hearing loss which can restore the patient’s ability to understand speech [1,2]. The cochlear implant (*CI*) works by directly stimulating the auditory nerve. This is accomplished by inserting a cochlear implant electrode array into the patient’s cochlea. An electric field stimulus is then applied by a number of contacts distributed along the electrode array, targeting the spiral ganglion cells and auditory nerve fibers.

For cochlear implantation, various types of electrode arrays from different manufacturers are available. These electrode arrays differ in size, length, number of electrode contacts and material characteristics [3]. Prior to surgery, a decision must be made by the patient and physician on which electrode to implant. To do so, multiple factors must be taken into account including the residual hearing and medical history of the patient, which may include otosclerosis, patient preference as well as the length and shape of the cochlea. The cochlear length is known to have large variations [4–22]. Previous studies have shown that for patients who only hear with their *CI*, improved outcomes after CI surgery can be expected with longer electrode arrays and accordingly deeper insertion angles [23–25]. Other studies have shown that the insertion angle not only depends on the electrode array type but also on the length of the cochlea [26,27]. Furthermore, dysplasia and other syndromes exist which have an effect on cochlear geometry, especially regarding the length, shape and number of turns (e.g. Mondini dysplasia) [28].

Different methods are available for the evaluation of the cochlea duct length (*CDL*) and the depth of insertion within clinical imaging data of cochlea without malformation: one option is to manually trace the contour of the cochlea or electrode array and subsequently use spline interpolation to determine the corresponding length [15,29]. Other methods, which are based on mathematical correlations, use the basal diameter A (within a logarithmic equation) to estimate the cochlea or array length [30–32]. The benefit of the latter is that these types of estimates do not require special software tools but can be employed using common DICOM viewers. However, if these estimations are used for patient specific considerations on which *CI* array to use, the corresponding *CDL* values must be accurate and reliable. In order to address both the impact of cochlear length variations onto cochlear implant surgery as well as the reliability of popular literature approaches to assess this variability, the proposed study was conducted.

Based on a large dataset of imaging data of *CI* patients, evaluations were performed on the distribution of *CDL* values, the correlation of electrode array length and the length of the cochlea covered by the respective array and suitability of specific *CI* arrays for certain ranges of *CDL* values. Following up on the study of Rivas et al. [33] who addressed the impact of A-value assessment deviations onto the electrode choice, it was further evaluated to which extent inaccuracies of the A-value method itself [30] would have led to a different choice of CI array than the respective contour tracings.

## Materials and methods

### Ethics statement

The ethics committee of the Hannover Medical School, Germany, approved this retrospective study. Due to the retrospective design, no written information was given to the patients of the study group. All patient data were anonymized and de-identified prior the retrospective analysis.

### Subjects

**Fig 1:**
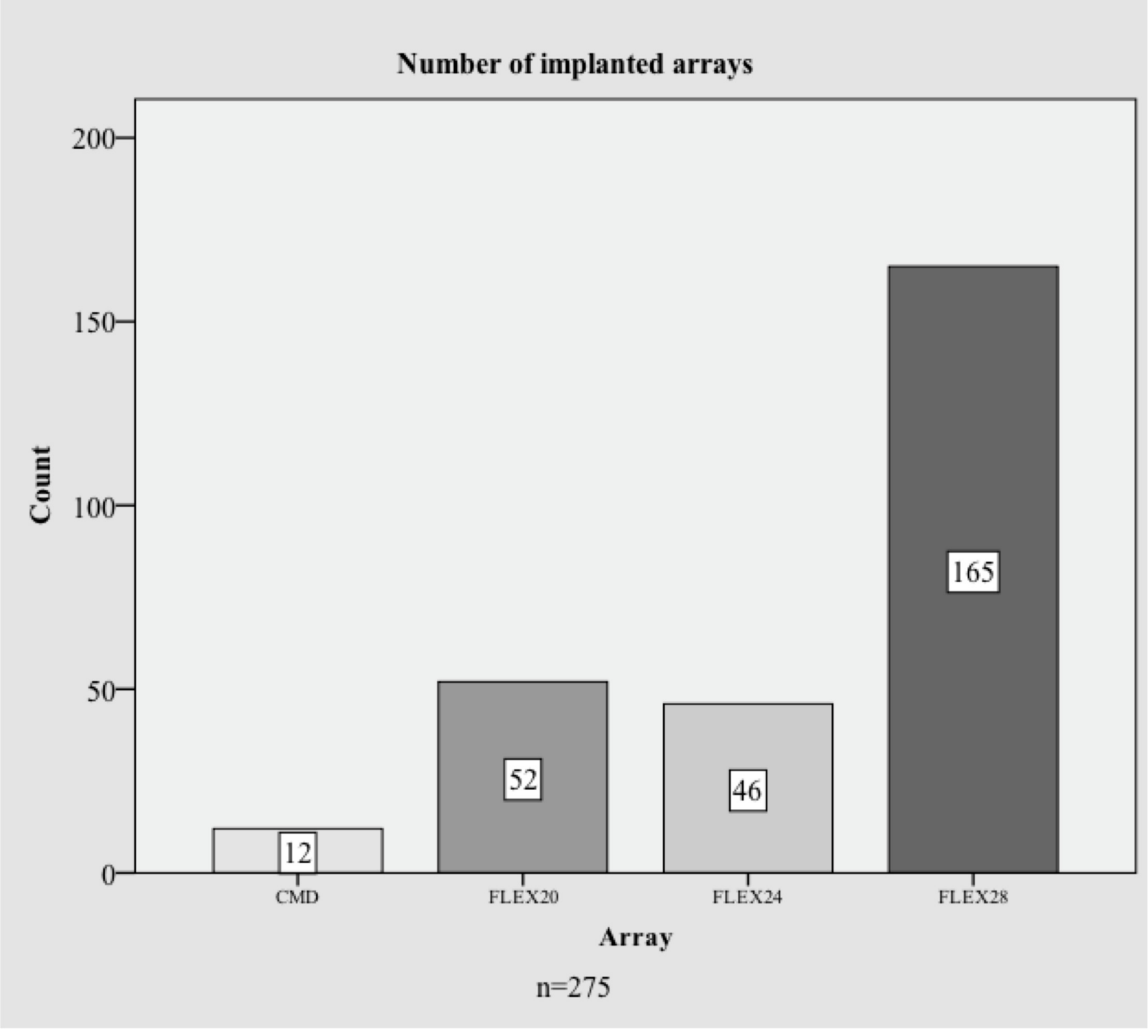
Study case overview. Overview of implanted electrode arrays which were included into this study. Note that the *CMD* group consists of FLEX^16^ electrode arrays and partial insertions of FLEX^24^ and FLEX^28^ electrodes).

At the Hannover Medical School, pre- and postoperative imaging of all *CI* patients is obtained by either Cone Beam CT (CBCT) or standard CT scans. We performed a retrospective study of 272 preoperative imaging datasets of patients who were implanted with a MED-EL FLEX^20^, FLEX^24^ or FLEX^28^ electrode between 2006 and 2017. Postoperative scans were available in 259 of these cases. Furthermore, patients who received custom-made devices (*CMD*) of 16mm length as well as 16 mm partial insertions of FLEX^24^ and 20 mm insertions with the FLEX^28^ were evaluated. Partial insertions were performed in an attempt to preserve residual hearing which were all considered *CMD* for the purpose of data analysis. Within the overall study group, most patients were implanted with a MED-EL FLEX^28^ (165 patients), 46 patients with a FLEX^24^, 52 patients with a FLEX^20^ and 12 patients with a MED-EL Flex *CMD* (see Fig 1). In our practice, pre- and postoperative imaging are a routine part of clinical care analyses.

### Imaging data analysis

**Fig 2:**
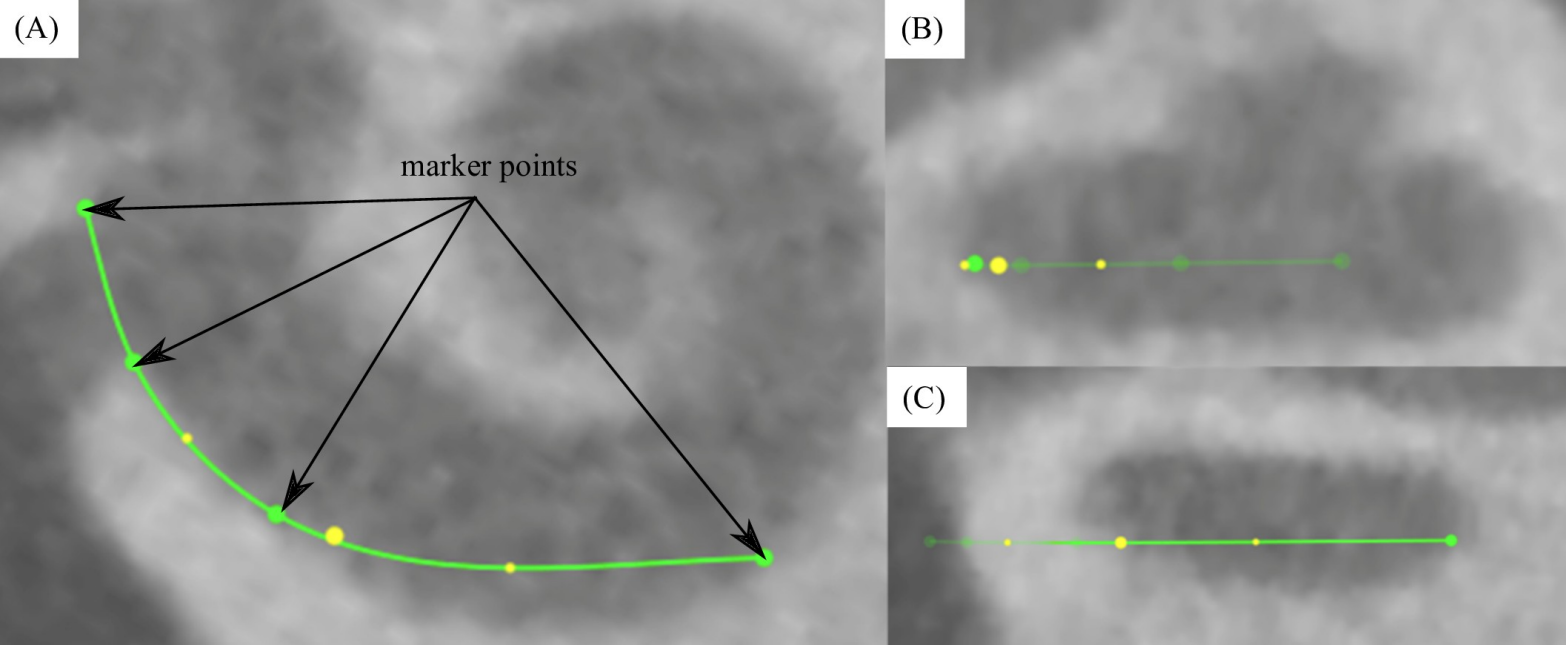
Spline measurement with OsiriX MD. Visualization of lateral wall tracing in OsiriX MD in top (A) and side views (B, C).

**Fig 3:**
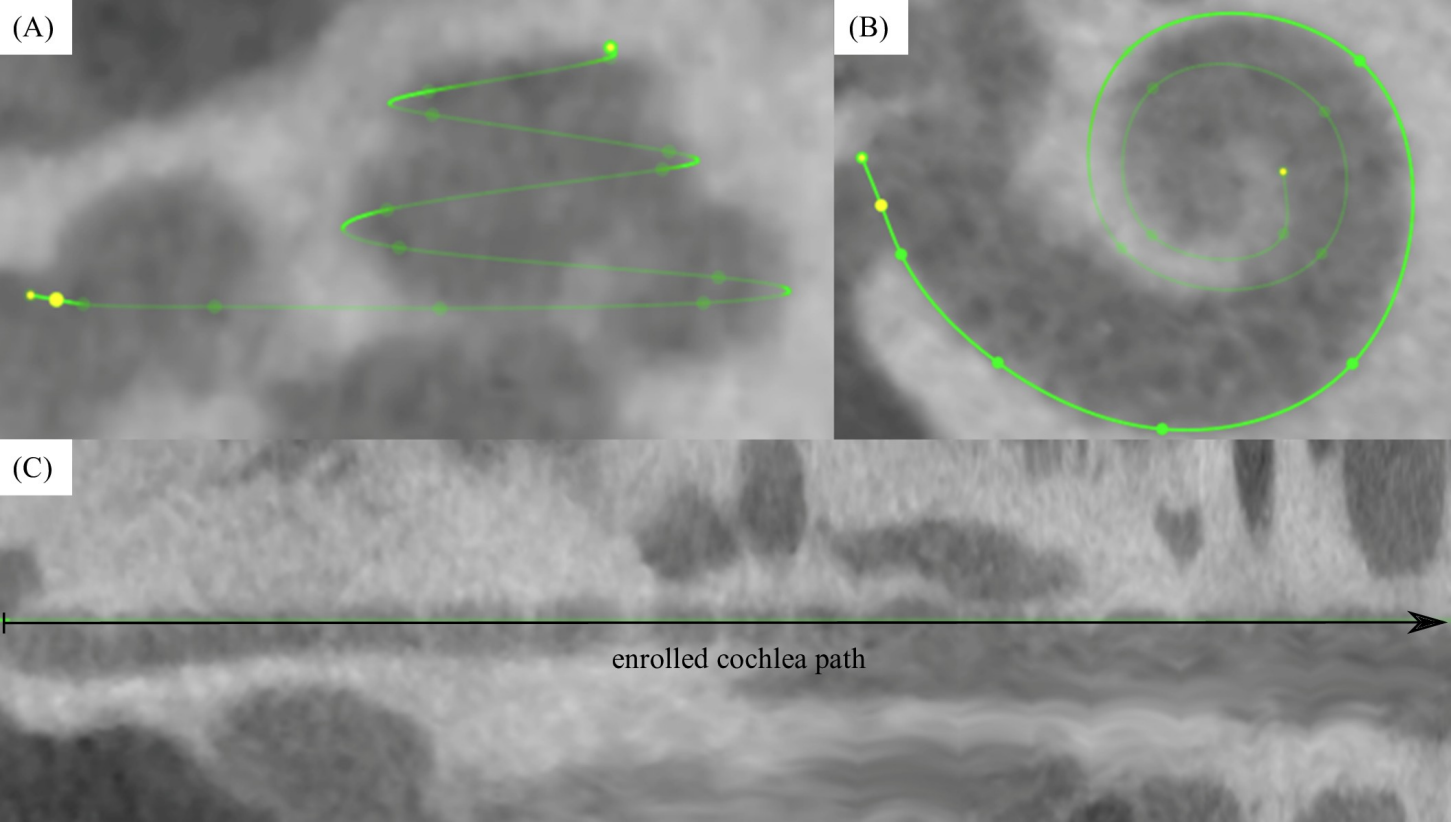
Complete segmentation of the lateral wall. Visualization of a completed lateral wall tracing in OsiriX MD (A, B) with the additional display of the enrolled path (C) for which the length is computed automatically.

**Fig 4:**
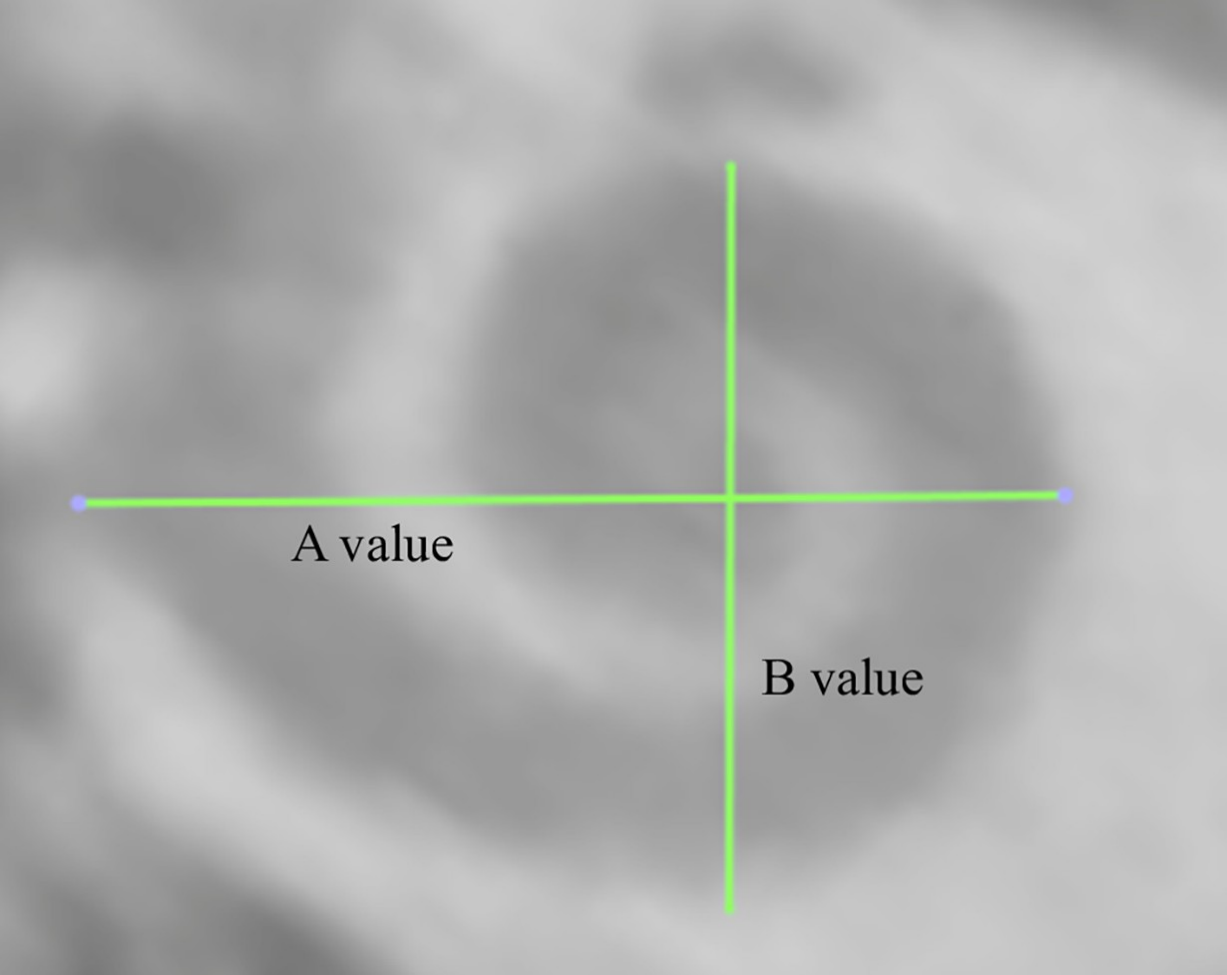
Measurement of global cochlea dimensions. Visualization of measurements of A (defined as the distance from the round window through the modiolus to the opposite wall of the cochlea) and B (maximal distance orthogonal to A).

**Fig 5:**
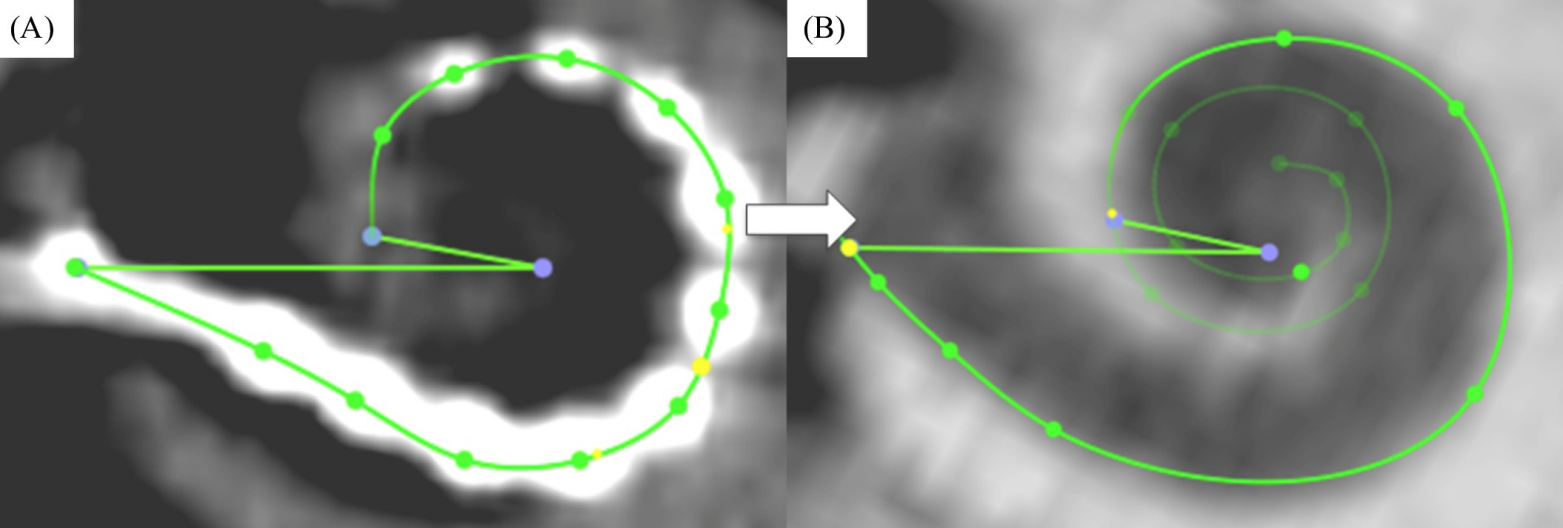
Inclusion of postoperative imaging analysis. (A) Postoperatively, marker points were placed in the center of the round window and along the inserted electrode array in the middle of the respective contact artefacts, and the corresponding insertion angle was measured. (B) The latter value was then used within the corresponding preoperative imaging data to determine the length along the lateral wall up to the insertion angle.

All datasets were evaluated using the DICOM-Viewer OsiriX MD (version 2.5.1 64bit, Pixmeo SARL, Switzerland). The corresponding analysis included the following steps:

- Tracings of the cochlear lateral wall from the center of the round window to the apex yield the corresponding *CDL* (see Figs 2 and 3)[15]. This is an automated feature within OsiriX MD. Performing one of these measurements takes approximately 2 minutes. An example is given in the supplementary material of this manuscript (see S1 Video).
- Measurements of the basal turn diameter *A* as well as the cochlear angle *(CA)* (see Fig 4).
- Measurement of the insertion angle (*IA*), defined as the angle from the center of the round window to the most apical contact (see Fig 5).
- Tracings of the cochlear lateral wall in the preoperative scan (in order to avoid inaccuracies due to artifacts of the implanted array, see Fig 5) from the center of the round window to the insertion angle, yielding the covered cochlea length (*CCL*).
- Measurement of the inserted electrode length (*IEL*) by placing marker points in the centers of all 12 electrode contacts as well as the entrance point of the electrode array in the round window (see Fig 5).
- Computation of the individual cochlear coverage (*CC*) in percent by dividing the corresponding *CCL* by the respective *CDL*.

### Measurement data analysis

After measurement data was acquired according to the methodology stated above, data analysis regarding the *CDL* and its estimation was performed in the following manner:

- Estimation of the cochlea length using the estimation method of Escudé et al. [30] by using *A* and the average *CA* value of 900 deg (or 2.5 turns):

$$CDL=2.62\;A\;ln\;\left(1+\frac{CA}{235}\right)$$

- Comparison of the measured *CDL* values derived by the lateral wall tracings and the ones estimated using the above equation.

Results regarding the coverage of the cochlea with specific electrode arrays, corresponding anatomical indication ranges for each electrode array and clinically relevant evaluation errors were derived as follows:

- Correlation of *IEL* and *CCL* in order to derive the relation between the length of the inserted electrode and the covered length of the cochlea.
- Determining the *CDL* indication ranges for the different electrode arrays was based on (a) the previous correlation and (b) the manufacturer’s recommendation of 80% cochlear coverage (*CC*) [34].
- Evaluating the accuracy of Escude’s method in terms of estimated *CDL* values suggesting the same CI array indication as the corresponding lateral wall measurements.

### Statistical analysis

The data was statistically analyzed using IBM SPSS Statistics (Version 24.0.0.0). To test whether a normal distribution exists we used the Kolmogorov-Smirnov test.

## Results

**Fig 6:**
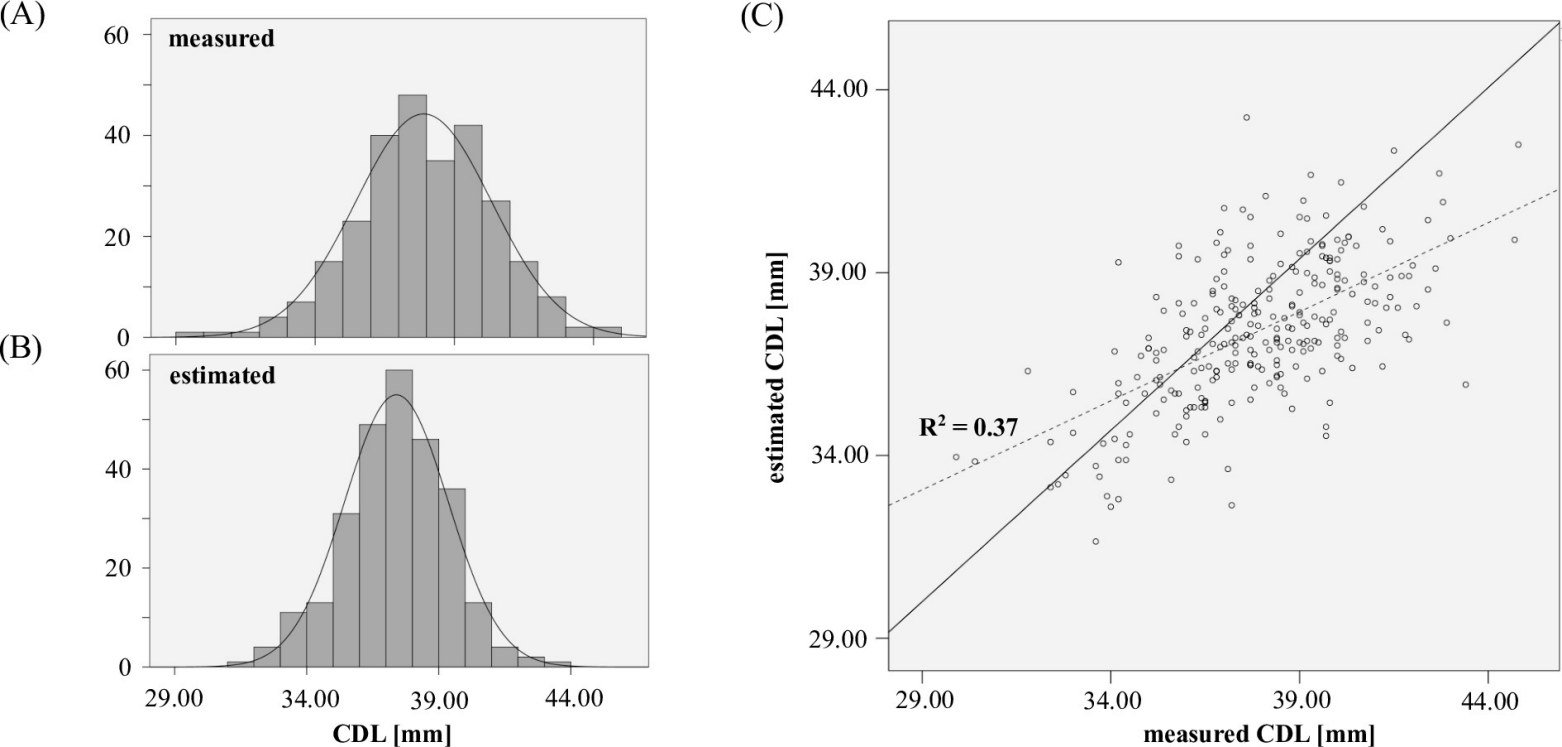
CDL distribution. Histograms of (A) measured and (B) estimated *CDL* values. (C) comparison of individual *CDL* measurements and estimations.

All measurement data can be found in S1 Table. Fig 6A and 6B show histograms of both measured and estimated *CDL*s of the 272 cochleae: In both cases a normal distribution was found with a mean length of 37.9mm and standard deviations of 2.4mm and 2.3mm respectively. The actual deviations of measurements and estimations are shown in Fig 6C: while the general trend of estimations and measurements is in agreement (R^2^ = 0.37), the mean absolute deviation was found to be at 1.4mm +/-1.1mm with a maximal deviation of 6.8mm.

**Fig 7:**
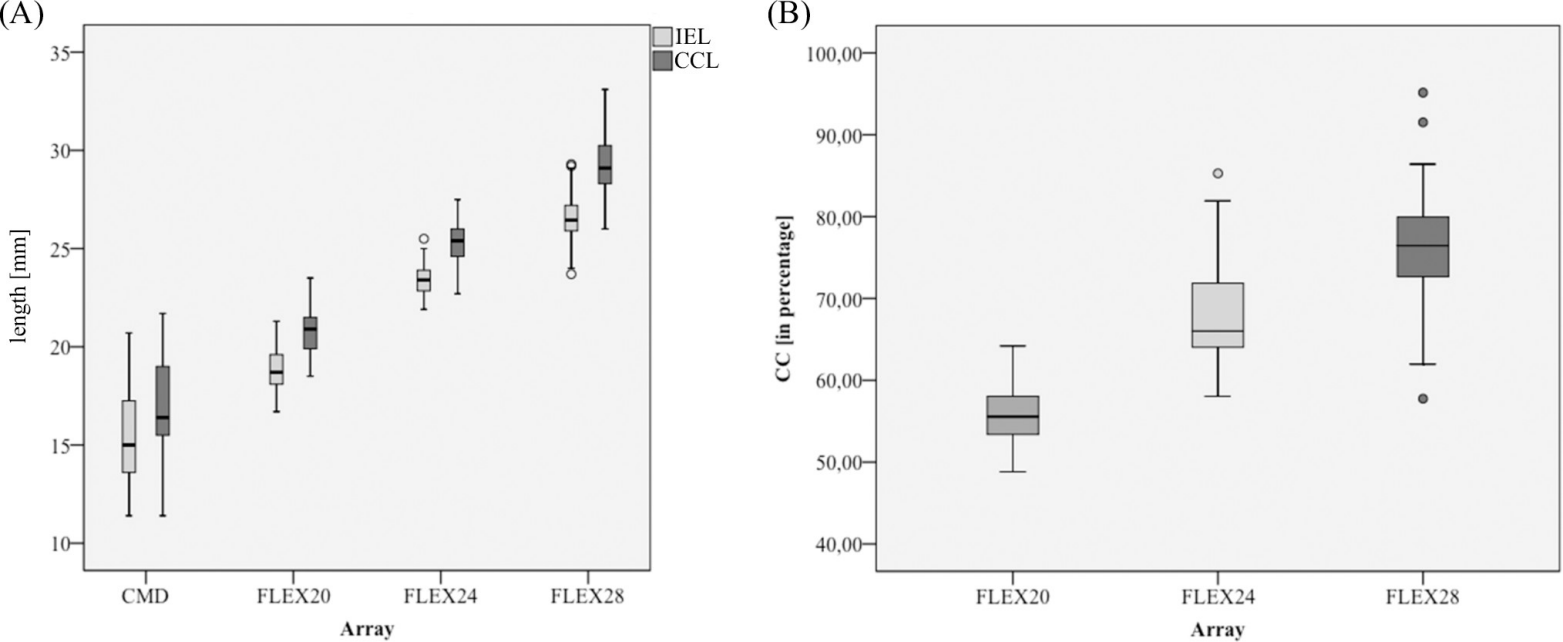
Distribution of electrode specific CI outcomes. (A) *IEL* and *CCL* distribution as well as (B) CC ranges for the different electrode array groups.

In order to evaluate the clinical relevance of these deviations, the postoperative measurement data was included into the analysis. Note that no tip fold over could be observed in any of the reviewed cases. Fig 7A shows the derived *IEL* and *CCL* values in a boxplot, grouped by the respective electrode array type. Means and the standard deviations of the *IEL* for the different arrays are 15.4mm +/-2.7mm for the *CMD* group, 18.7mm +/- 1.1 mm for the FLEX^20^, 23.5mm +/- 0.9 mm for the FLEX^24^ and 26.6mm +/- 1.1mm for the FLEX^28^. Mean values and standard deviations of the *CCL* are slightly larger (about 2 mm on average) than the respective *IEL* values with 20.8mm +/- 1.1mm for the FLEX^20^, 25.2mm +/- 1.2mm for the FLEX^24^ and 29.2mm +/-1.4mm for the FLEX^28^. The mean *CCL* of the *CMD* group was found to be 17mm +/- 2.9mm. Taking the preoperatively measured *CDL* into account, the individual cochlear coverage (*CC*) of the lateral wall can be calculated as the ratio of *CCL* and *CDL*, which is shown in Fig 7B. The achieved mean *CC* is 56% +/-3.5% for the FLEX^20^, 67.9% +/-6.1% for the FLEX^24^ and 76.4% +/-5.3% for the FLEX^28^. For the *CMD* devices a coverage of 46% +/- 7.6% was achieved. The *CC* variation for the longest electrode (FLEX^28^) ranged between 57.8% and 95.2% while it ranged from 58–85.3% for the FLEX^24^ and from 32.6–43.2% for the FLEX^20^.

**Fig 8:**
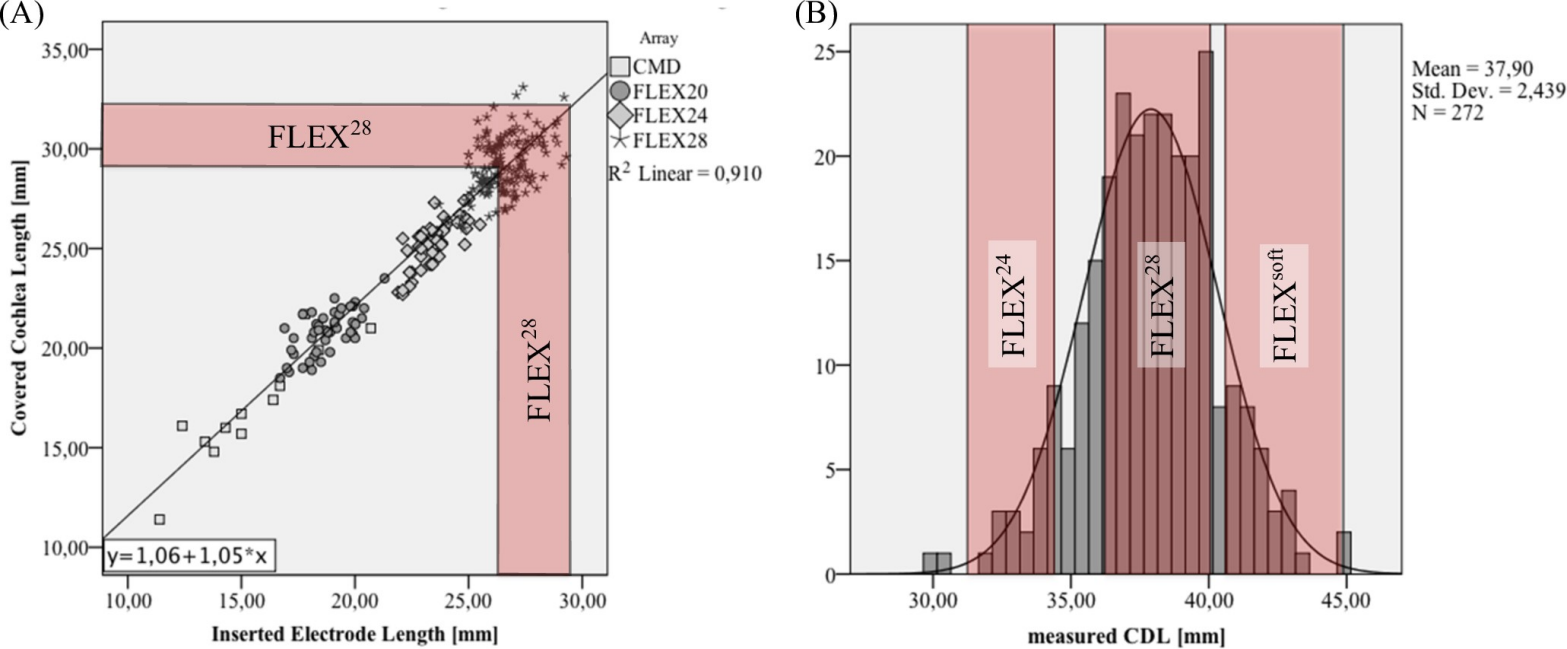
Anatomical indication ranges. (A) Depiction of the CCL range computed for a fully inserted (+/-5%) FLEX^28^ array, (B) visualization of the corresponding CDL indication ranges for FLEX^24^, FLEX^28^ and FLEX^Soft^ electrode arrays.

In order to correlate *IEL* and *CCL*, a scatter plot of the two is given in Fig 8A. Linear regression of these data points yielded the following correlation function:

CCL=1.06+1.05*IEL

The red area within the graph indicates (based on the example of a FLEX^28^ electrode array) the *CCL* range which can be expected for a successfully implanted array, if successful insertion is assumed to lie within +/- 5% of the array length that is supposed to be implanted (i.e. 28mm for a FLEX^28^ electrode array), the correlation function above can be employed to derive the corresponding *CCL* range. Table 1 shows the corresponding *IEL* and *CCL* ranges for different MED-EL electrode arrays suitable for patients without residual hearing. As mentioned before, the manufacturer recommends 80% *CC* (Mistrík & Jolly, 2016). Thus, translating the C*CL* to a clinical indicated range can be accomplished by dividing the derived CCL ranges by 0.8. The corresponding indicated ranges for the different arrays are listed in Table 1 and depicted in Fig 8B. Note that the indicated range for the FLEX^28^ array matches the peak of the derived *CDL* distribution.

**Table 1:** Indication range for the FLEX electrodes.

Array	FLEX 24	FLEX 28	FLEX Soft
**Array-length in mm**	24	28	31,5
***CCL* in mm**	26.3 (25–27.5)	30.5 (29–32)	34.2 (32.5–35.9)
**Recommended *CDL (*lateral wall) in mm**	32.9	38.2	42.7
**Indicated CDL (lateral wall) ranges (mm)**	31.3–34.4	36.2–40.1	40.6–44.9
**Recommended organ of Corti length (mm)**	26.5	30.5	34
**Indicated organ of Corti range (mm)**	25.3–27.7	29.1–32	32.4–35.7

**Fig 9:**
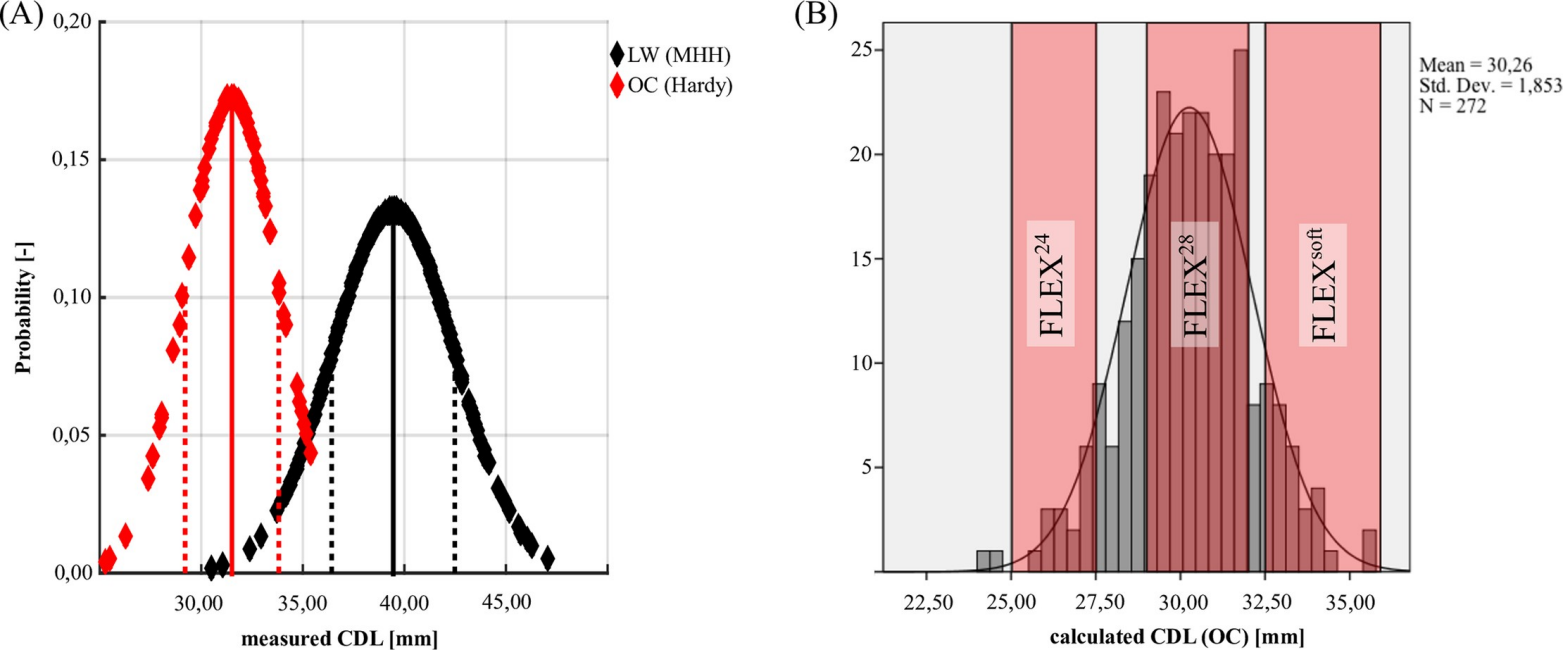
Organ of Corti length. (A) Histogram of the organ of Corti (OC) derived by Hardy and Lee (red) and MHH data (black), (B) Histogram of calculated OC length for MHH data with electrode lengths for FLEX^24^, FLEX^28^ and FLEX^Soft^ (+/- 5%).

Several publications refer to the *CDL* as the length of the organ of Corti (*OC*) and not the lateral wall. That is why the data of Hardy and Lee [5,31,35] was used to project the lateral wall indication ranges onto the organ of Corti (see Fig 9A). Linearly relating the *CDL* mean values +/- one standard deviation for lateral wall (37.9 mm +/- 2.4 mm) and organ of Corti (31.5 mm +/-2.3 mm) yielded the following equation:

$${CDL}_{OC}=0.76*{CDL}_{Lw}+1.46$$

This equation was used to translate the lateral wall indication ranges into the ones for organ of Corti, which are displayed in Fig 9B and stated within the last two rows of Table 1.

**Fig 10:**
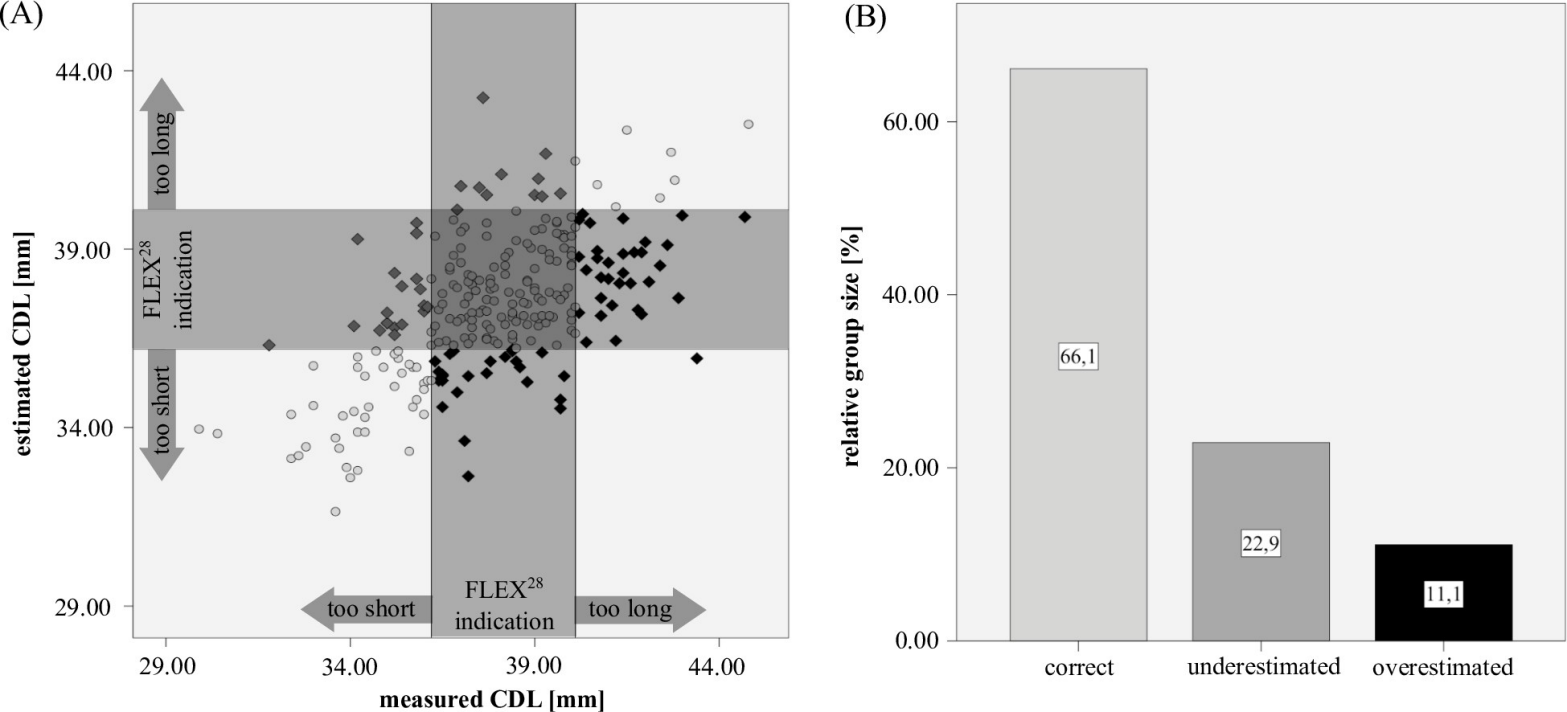
CDL identification of FLEX^28^ candidates. (A) the comparison of measurement and estimation-based identifications of FLEX^28^ candidates resulted in (B) correct identifications in 66% of the analyzed anatomies using Escudé’s method.

Finally, we evaluated if *CDL* estimations using the *A* value and *CA* [30] could be used instead of actual measurements to derive an accurate anatomical indication for a specific array. Note that only the length of the cochlea and not the length of the implanted array were taken into account for this analysis. Using the FLEX^28^ implanted group we then analyzed to what extent the *CDL* estimation method would predict cochlear anatomies as too short, applicable or too long for this array. Within Fig 10A, the x-axis represents the measured *CDL* whereas the y-axis shows the corresponding estimations. The shaded areas within this graph represent the FLEX^28^ indication ranges for measured and estimated CDL values respectively (3^rd^ column of Table 1). Correct identification, i.e. a match of measured and estimated indication (highlighted in the table), of short cochleae could be achieved in 67.8%, of long cochleae in 17.1% and of matching cochleae in 77.2% of the cases (see Table 2 and Fig 10B).

**Table 2:** Correct identification of cochlea length.

	Reference small	Reference match	Reference large	Correct Identification
**Estimation large**	0	11	**7**	7
**Estimation match**	19	**132**	33	132
**Estimation small**	**40**	28	1	40
**total number**	59	171	41	271
**correct identification in percentage**	67.8	77.2	17.1	66.1

## Discussion

In our study, we analyzed the anatomy of the cochlea in 272 clinical imaging datasets. The postoperative location of different lateral wall electrode arrays could be assessed in 259 of these 272 cases. The derived variability of the cochlea length is in agreement with previous studies [4,5,15–17] and highlights again the importance of considering the patient specific anatomy in the field of cochlear implantation (see Fig 6A).

The projection of the wide portfolio of available electrode arrays [3] onto this range of cochlear length values then allows for optimal coverage of the intracochlear neural structures: achieving 80% *CC* with lateral wall electrodes in case of an average or long cochlea, for instance, would currently only be achievable with MED-EL devices who also offer electrode array lengths of more than 26mm. However, it was not yet clearly proven if electrode arrays stimulate the neural fiber endings at the organ of Corti or the spiral ganglion cells directly, i.e. what cochlear coverage achieves the best possible speech perception. Nevertheless, recent publications show superior speech understanding for the FLEX^28^ array (which typically achieves insertion angles of 540–720 degrees) in comparison to shorter electrode arrays [24]. The derived indication ranges yield a possible explanation for this finding, i.e. the sufficient coverage of neural structures for most cochleae with the FLEX^28^ array.

The comparison of measured and estimated [30] *CDL*s showed normal distributions in either case with no significant differences, but quite severe deviations were found for individual cases (see Fig 6C) with deviations of up to 6.8mm. Hence, the *CDL* estimation method described by Escudé et al. [30] may be applicable for retrospective analysis but is not suitable for clinical use where accuracy matters for each and every individual case. This was further highlighted in Fig 10B where the estimation deviations were evaluated in terms of anatomical indications for specific CI electrode array lengths: the results show that in total, correct indications could only be derived in 66% of the analyzed cochleae. Especially concerning are the 11% of the cochleae whose *CDL* values were overestimated: in a clinical setting, overestimations could result in implantations of electrode arrays too long for a specific cochlea such that the array cannot be fully inserted and/or possibly cause intracochlear damage. Underestimations, on the other hand, were found to occur in 22% of the cases and could entail insufficient coverage of the cochlear neurons and hence result in poor speech understanding, since studies show that longer electrodes result in a better speech understanding for electric stimulation only [23,24].

The *IEL* and corresponding *CCL* values for the analyzed electrode arrays are shown in Fig 7A. *CCL* values are larger than the according *IEL* values for each electrode array due to the latter being located in closer proximity to the modiolus than the lateral wall and thus representing a shorter distance. The projection of the array from the modiolus out onto the lateral wall hence creates a larger spiral profile and an accordingly larger *CCL* value. The group of *CMD*s shows the highest deviation regarding the *IEL* to *CCL* correlation. This fact is owed to the *CMD* group containing FLEX^24^ and FLEX^28^ arrays which were only partially inserted (covering only the individual frequency region with severe to profound hearing loss) as well as custom made devices of 16mm length.

While *CC* increases for longer electrodes overall (see Fig 7B), it can be seen that the *CC* distributions overlap especially regarding the FLEX^24^ and FLEX^28^ arrays. This can be explained by the fact that the *CC* is not only dependent on the length of the electrode array but also on the length of the cochlea [27]. In some cases, a FLEX^24^ in a short cochlea may therefore cover a larger fraction of the cochlea than a FLEX^28^ in a large cochlea. It should further be noted that the smallest *CC* values can be found within the FLEX^20^ group because this electrode was developed to not cover the full frequency range of the cochlea in order for patients to benefit from acoustic stimulation in the low frequencies using an EAS system.

Variations in *CC* values generated with the same array can also be seen in Fig 8A. Although the overall cochlea length does not directly influence *CCL* values, it can be assumed that the same kind of array will be situated differently in a small cochlea than in a large one. Variations in cochlear anatomy are therefore likely to also influence the length of the cochlea covered by an electrode array.

**Fig 11:**
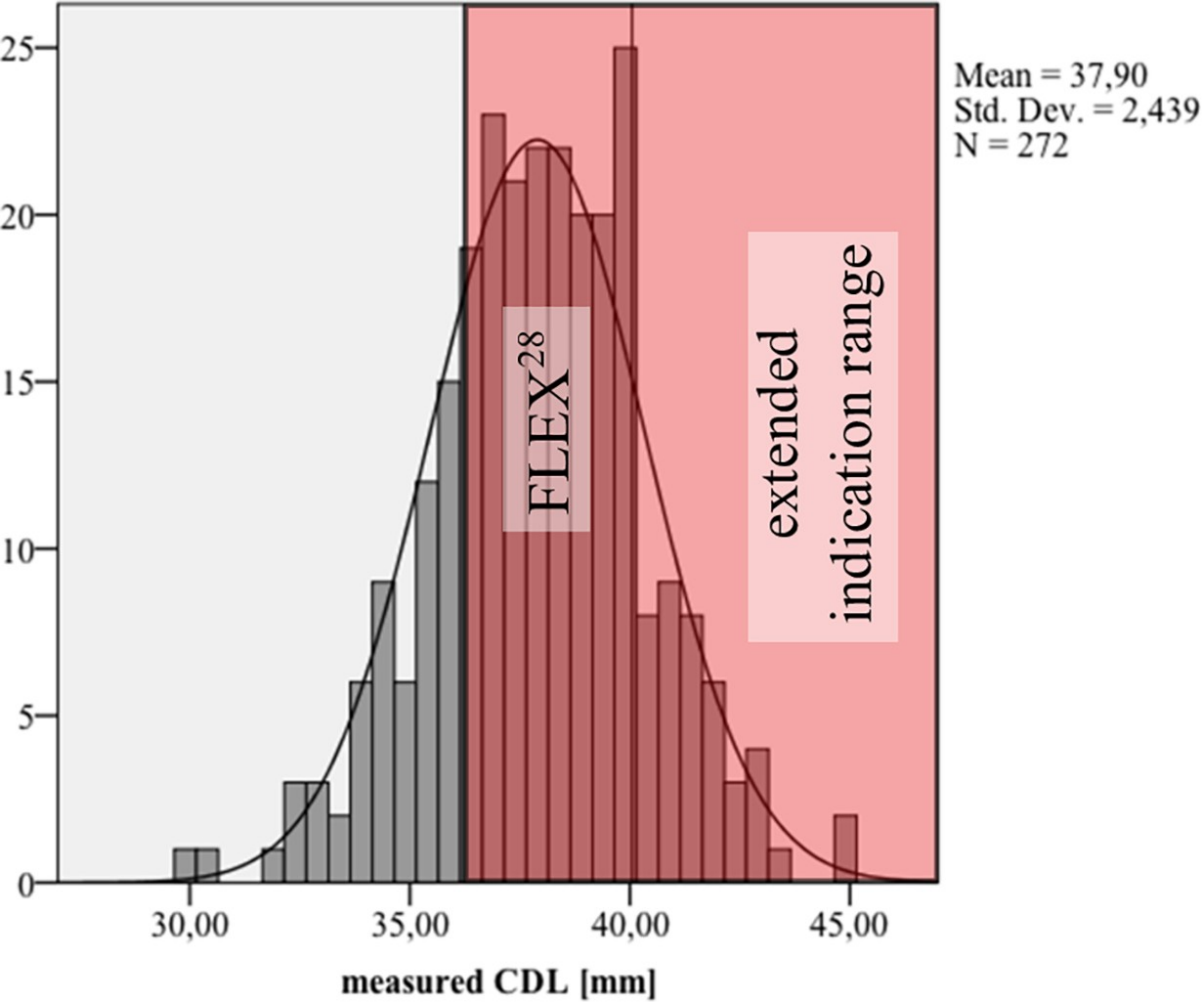
Extended indication range for MED-EL FLEX^28^. Structure preservation indication range for the FLEX^28^ array which could have been fully inserted in 76% of the investigated cases.

Regarding the anatomical indication ranges it was found based on the measured *CDL* that most cochleae are covered by the FLEX^28^ with a target *CDL* range of 36.2mm to 40.1mm (11+132+28 of the 271 cases corresponding to 63% of the analyzed cases). Without knowing the anatomy, a FLEX^28^ electrode also seems to be the best choice since it could be implanted in 76.8% (see Fig 11) of the cases with a reduced risk of harming any intracochlear structures.

## Conclusion

Following previous investigations, we showed that due to the variation of cochlear size, preoperative cochlea length assessment should be included into the routine preoperative care. Furthermore, individual implant selection based on the patient specific cochlea length is feasible and likely to improve implantation outcomes. However, length estimations exclusively based on mathematical correlations may result in false recommendations and could potentially injure the cochlea and lead to poorer outcomes. Furthermore, the presented results demonstrate that most cochleae are sufficiently covered by a FLEX^28^ electrode array while the FLEX^24^ and FLEX^Soft^ should be employed for short and long cochleae respectively.

## Supporting information

S1 Video Video of CDL measurement in preoperative data. Preoperative measurement of a cochlear duct length (CDL) in a Cone Beam CT scan (CBCT).
(MOV)

S1 Table Measurement data of the study. (XLSX)
